# Problematic Smartphone Use: An Overlooked Trigger for Oculomotor Strain, Cervical Disability, and Headache in Medical Students

**DOI:** 10.14789/ejmj.JMJ25-0038-OA

**Published:** 2025-12-18

**Authors:** NOOR AHMED SHAH SYED, JUNAID MAJEED, CHINENYE IGUH, MANOHARAN DHIVAHARAN, SAHAR YASEEN, FATIMA JABER, GYANENDRA K.C., TAMUNONENGIYEOFORI ESTHER SOFIMIEARI, HINZA HASSAN, SARA LIBA CHANGAAI MANGALOTE, RACHA ALNIAZI, AUROOBA NAEEM

**Affiliations:** 1 Department of Internal Medicine, Liaquat University of Medical and Health Sciences, Jamshoro, Sindh, Pakistan; 1 Department of Internal Medicine, Liaquat University of Medical and Health Sciences, Jamshoro, Sindh, Pakistan; 2 Department of Medicine, King Edward Medical University, Lahore, Pakistan; 2 Department of Medicine, King Edward Medical University, Lahore, Pakistan; 3 Department of Medicine, Windsor University School of Medicine, Chicago, Illinois, USA; 3 Department of Medicine, Windsor University School of Medicine, Chicago, Illinois, USA; 4 Department of Internal Medicine, University of Perpetual Help System DALTA, Las Piñas, Manila, Philippines; 4 Department of Internal Medicine, University of Perpetual Help System DALTA, Las Piñas, Manila, Philippines; 5 Department of Internal Medicine, Royal Blackburn Hospital, Manchester, UK; 5 Department of Internal Medicine, Royal Blackburn Hospital, Manchester, UK; 6 Department of Medicine, Allama Iqbal Medical College, Lahore, Pakistan; 6 Department of Medicine, Allama Iqbal Medical College, Lahore, Pakistan; 7 Department of Internal Medicine, KIST Medical College & Teaching Hospital, Imadol, Lalitpur, Nepal; 7 Department of Internal Medicine, KIST Medical College & Teaching Hospital, Imadol, Lalitpur, Nepal; 8 Department of Internal Medicine, Nottingham University Hospital NHS Trust, Nottingham, UK; 8 Department of Internal Medicine, Nottingham University Hospital NHS Trust, Nottingham, UK; 9 Department of Internal Medicine, Dubai Medical College, Dubai, UAE; 9 Department of Internal Medicine, Dubai Medical College, Dubai, UAE; 10 Department of Orthodontics, Dubai Health, Dubai, UAE; 10 Department of Orthodontics, Dubai Health, Dubai, UAE; 11 Department of Clinical Psychology, Shifa Tameer-e-Millat University, Islamabad, Pakistan; 11 Department of Clinical Psychology, Shifa Tameer-e-Millat University, Islamabad, Pakistan

**Keywords:** problematic smartphone use, computer vision syndrome, cervical disability, headache impact, medical students

## Abstract

**Objectives:**

Problematic smartphone use (PSU) is widespread among medical students and might be associated with visual, musculoskeletal, and neurological complaints. In this study, we investigated the relationship between PSU and oculomotor strain, cervical disability, and the impact of headaches.

**Materials and Methods:**

The present study was conducted through a cross-sectional survey among 498 medical students in Lahore, Pakistan. PSU was assessed using the Smartphone Addiction Scale-Short Version (SAS-SV), oculomotor strain with the Computer Vision Syndrome Questionnaire (CVS-Q), cervical disability with the Neck Disability Index (NDI), and headache impact using the HIT-6. Data were assessed using Pearson's correlations, t-test, ANOVA, and multivariate regression in SPSS v.26.

**Results:**

The average SAS-SV score was 32.1 ± 4.9. PSU was associated with CVS (r = 0.229), NDI (r = 0.147), and HIT-6 (r =  0.088). Scores for all measures were higher among female students (p < 0.001). The youngest students presented with higher PSU; the CVS was more intense in 21-23 years of age, and older students had a greater cervical disability. Predictors of CVS, cervical disability, and headache impact were PSU, gender (female), younger age, low physical activity status, and comorbidities according to the regression models.

**Conclusion:**

PSU is associated with increased oculomotor strain, neck disability, and headache burden in medical students, especially for females, younger-aged students, and those having low activity or comorbidities. Preventive strategies that promote digital well-being and healthy behaviours are advised.

## Introduction

Smartphones have become an essential device in students' daily lives; the phones used for education, as well as social and entertainment purposes, are now recognised as a portable mobile learning tool^1, 2)^. Problematic smartphone use (PSU), characterised by excessive and uncontrolled use of smartphones that causes damage to an individual's functioning and has negative consequences, occurs in approximately 23% of children and youth^3, 4)^.

Problematic smartphone, social media, and internet use were associated with poor health outcomes in medical students, supporting the relevance of digital wellbeing in student welfare initiatives^5)^. Prolonged smartphone and tablet use over time, both daily, is related to symptoms of eyestrain, headaches, as well as musculoskeletal complaints, indicating that protracted device-related physcomotor effects may add visual and physical risk factors^6, 7)^.

Digital eye strain (DES), a condition characterised by visual disturbances and ocular discomfort resulting from prolonged use of digital devices, affects up to 90% of individuals^8)^. Research has indicated that 86% of smartphone users experience at least one symptom of DES, with the highest prevalence rates for eye fatigue (53%), blurred vision (39%), and ocular discomfort (34%), while greater daily smartphone use results in increased severity of symptoms^9)^.

Neck disability is also common in students with problematic smartphone use. Smartphone addiction was found in 83% of the students, and mild neck disability among 50%^10)^. Headache disorders are estimated to affect almost 90% of the population, and primary headaches, including migraine and tension-type headache, are the most prevalent^11)^. Smartphones have been linked to greater severity of headaches and higher demand for acute medications, as users reported less effective treatment compared with non-users^12)^.

In light of these associations, this study proposes to explore the relationship between PPSU and its impact on oculomotor strain, neck functional disability (NFD), and headache burden among medical students. This paper aims to draw attention to the problem and present evidence that supports the adoption of preventive measures for promoting healthy digital habits among students.

### Rationale

Medical students use smartphones extensively for studying, communicating, and entertainment. Excessive or uncontrolled smartphone use may lead to physical issues, including headaches, eye strain, and neck pain, which can interfere with daily activities and academic performance. Investigating these potential health effects is crucial to understanding how our device use patterns impact students' overall health.

Despite increasing global evidence on PSU and its associated health disparities, little information is available about the Pakistani setting. This study, by examining these correlations among medical students, aims to contribute to the development of indigenous evidence that can be utilised for preventive interventions, sensitisation, and adoption of healthier digital practices among the academic population. The results may be helpful for the development of potential interventions to reduce physical and functional impairments resulting from smartphone overuse.

## Materials and Methods

### General objective

• To investigate the association of problematic smartphone use (PSU) and severity in patients experiencing oculomotor strain, cervical disability, and headache burden among medical students.

### Specific objectives

• To find out the problematic use of smartphones among Medical Students.

• To assess oculomotor stress related to the use of smartphones in this group.

• To evaluate the severity of cervical spine discomfort or disability associated with smartphone use.

• To assess the influence of smartphone-related behaviour on headache frequency and severity.

### Study design

A cross-sectional questionnaire-based study was conducted to investigate the relationship between problematic smartphone use and its impact on oculomotor strain, cervical disability, and headache burden among medical students. Students were enrolled from medical colleges in Lahore, Pakistan using convenience sampling to diversify the student body, which included students from various years of study and diverse socioeconomic backgrounds.

### Sampling strategy and population size

The sample size was computed using the World Health Organisation (WHO) formula to determine a population proportion at a 95% confidence level and 5% margin of error^13)^. Using a conservative anticipated proportion of 0.5, the sample size calculated for each group would be approximately 384. To account for some non-respondents and incomplete questionnaires, the study included more participants to achieve a sufficient result.

In all, 530 medical students were initially enrolled; of them, only 498 returned complete and valid questionnaires and were considered for the final analysis. This final sample size was above the minimum required, maintaining adequate power to investigate the association between problematic smartphone use and its associated oculomotor strain, cervical disability, and headache burden outcomes. The study employed a non-random convenience sampling approach, where all medical students present on the day of data collection from a selected institution were included in this assessment.

### Inclusion and exclusion criteria

The study sample consisted of undergraduate medical students from accredited medical colleges in Lahore who owned and regularly used smartphones. Only those who gave informed consent were eligible to participate. The students who had a prior history of neurological, ophthalmological, and musculoskeletal diseases unrelated to smartphone use and those with a history of head or neck trauma or surgery within the last 6 months were excluded from this study. Participants who refused to participate or submitted incomplete questionnaires were also excluded from the final analysis.

### Data collection tools

We developed a structured questionnaire to obtain an adequate and detailed set of data for the scope of this study, which was divided into four main parts: demographics, assessment of problematic smartphone use, ocular motor strain evaluation, and measurement of cervical disability and headache impact. All standardised measures were presented in English, which was appropriate for a general population sample that had complete command of the language, and no cultural/linguistic translations were necessary.

### Demographic form

The demographic part gathered key background information, such as age, sex, year of study, daily status of smartphone use, and lifestyle factors (study time and duration exposed to the screen). This information was used to characterise the study population and to detect differences in smartphone use patterns among participants.

### Smartphone addiction scale - short version (SAS-SV)

The SAS-SV was first developed by Kwon et al. in 2013 and has been utilised as a brief assessment instrument for identifying problematic smartphone use among adolescents and young adults. This scale comprises 10 items on a 6-point Likert scale, ranging from "strongly disagree" (1) to "strongly agree" (6), with total scores ranging from 10 to 60. A higher score represents a stronger attitude toward smartphone addiction, and cut-off values of 31 for males and 33 for females are recommended. The SAS-SV has demonstrated high internal consistency, with Cronbach's alpha ranging from 0.86 to 0.91. We used the SAS-SV to classify medical students into risk groups for problematic smartphone use^14)^.

### Computer vision syndrome questionnaire (CVS-Q)

The CVS-Q questionnaire was created in 2015 by Seguí et al. as a standardised instrument to quantify visual and ocular symptoms resulting from extensive use of digital screens. It includes 16 questions that assess how often and to what extent people experience symptoms, including eyestrain, blurred vision, tearing, dryness, and headaches. The frequency (ranging from "never" to "often/always") and intensity (moderate or severe) of each symptom are scored to establish a weighted index system for computer vision syndrome. The CVS-Q has demonstrated excellent internal consistency, with a Cronbach's alpha of 0.87, confirming its reliability as a helpful assessment tool for both clinical and research purposes. In the current study, CVS-Q was used to assess visual strain resulting from smartphone overuse among medical students^15)^.

### Neck disability index (NDI)

The NDI was first introduced in 1991 by Vernon and Mior as an adaptation of the Oswestry Low Back Pain Disability Index for measuring functional disability due to neck pain. It comprises 10 items, scored on a 6-point Likert scale ranging from 0 to 5, covering the domains of pain intensity, personal care, lifting, reading, concentration, work performance, driving, sleeping disturbances, recreational activities, and headaches. Scoring is based on a 0-to-5-point scale, where higher values indicate greater disability. Disability severity ranges from none (0-4) to mild (5-14), moderate (15-24), severe (25-34), and complete (≥ 35). The NDI exhibits good internal consistency, as evidenced by Cronbach's alpha scores ranging from 0.80 to 0.93. In the present investigation, NDI was employed to measure cervical disability associated with problematic smartphone use in medical students^16)^.

### Headache Impact Test (HIT-6)

The HIT-6 was developed by Ware, Kosinski, and others in 2003 as a brief instrument to assess the negative impact on quality of life caused by headaches. It is comprised of 6 questions that address pain, social and role functions, vitality, cognitive function, and emotional distress. The score of each item ranges from 6 to 13 on the 5-point Likert, with a total score ranging from 36 to 78. Greater scores indicate more effective influence of headaches: no/little impact (≤ 49); some impact (50-55); good/considerable impact (56-59), intense effect/severe effect (> 60). The HIT-6 also exhibits good internal consistency with a Cronbach's alpha of ~0.89^17)^. For medical students, the HIT-6 was used to assess the extent of headache-related impact on daily functioning in relation to problematic smartphone use.

### Procedure

The study was conducted on the students of medical colleges. We collected the data over four months, from June to September. Screened students who were found eligible as per the inclusion criteria were approached during their classroom teaching sessions/study time/clinical postings and told about the objectives, rationale, and design of the study to avoid any information bias. All information was gathered with written informed consent from participants. Privacy and confidentiality were particularly stressed; no names or other identifying data were collected, and responses to the survey were anonymous. This methodology enabled reliable data collection and an ethical process, and a supportive academic setting for participants.

### Analytical approach

IBM SPSS Statistics version 26 (IBM Corp.) was used to analyse the data. The demographic and background data of the participants were summarised using descriptive statistics, which included frequencies and percentages. Kolmogorov-Smirnov and Shapiro-Wilk tests were used to check the normality of the main study variables. The Pearson correlation coefficients were calculated to explore the relationships between smartphone addiction, computer vision syndrome, cervical disability, and the effect of headaches. Gender-based differences in the study outcomes were analysed with independent samples t-tests, and differences across age groups were analysed with one-way ANOVA. The graphical visualization of standardized regression coefficients was performed, and multiple regression analyses were used to determine the predictors of computer vision syndrome, cervical disability, and the impact of headache, including smartphone addiction, demographic, and behavioral covariates. Statistical tests were all two-tailed with a significance level of p < 0.05.

### Ethical protocols

The study was reviewed and approved by the Institutional Review Board of Allama Iqbal Medical College (038s-AIMU-IRB-2025). The study was conducted in accordance with the ethical principles of research involving human subjects (Declaration of Helsinki). Participants' dignity and rights were promoted by ensuring that failure to participate or withdrawal from the study would not affect their academic status. To maintain scientific validity, questionnaires with incomplete or invalid data were excluded from the final analysis.

## Results

### Demographic, lifestyle, and health-related characteristics of participants

Table 1 shows demographic and health-related data of 498 medical students. Most of the respondents were women (N = 288, 58%), and they were aged 18-21 years (N = 350, 70%). The majority of students experienced moderate levels of neck disability (N = 405, 81%), and substantial or severe levels of headache impact (N = 160, 32%, and N = 108, 22%, respectively). In terms of lifestyle habits, 208 students (42%) were physically active 1-2 days per week, and 198 students (40%) slept 5-6 hours per night. Self-reported medical problems were most frequently eye-related (N = 148, 30%), with the most significant proportion being either contact lenses (N = 153, 31%) or both spectacles and contact lenses (N = 215, 43%). The primary internet use device among 210 students was a smartphone (42%), and 5-8 hours of daily internet connectivity was most prevalent (N = 210, 42%). All in all, these data demonstrate that the sample is primarily young and is composed of females with moderate musculoskeletal and ocular load and intensive use of digital devices.

**Table 1:** Demographic characteristics of participants (N = 498)

Variable	f (N)	%		Variable	f (N)	%
CVS positive (CVS-Q ≥ 6)				Spectacles/contact lens use		
No	180	36		Yes, spectacles	43	9
Yes	318	64		Yes, contact lenses	153	31
NDI severity				Both	215	43
Mild	3	1		No	87	17
Moderate	405	81		Average sleep duration (per night)		
Severe	84	17		Less than 5 hours	89	18
No disability	6	1		5-6 hours	198	40
HIT severity				7-8 hours	172	34
Little/No impact	90	18		More than 8 hours	39	8
Some impact	140	28		Average physical activity (per week)		
Substantial impact	160	32		None	45	9
Severe impact	108	22		1-2 days	208	42
Age				3-4 days	120	24
18-21 years	350	70		5 or more days	125	25
21-23 years	75	15		Existing medical conditions (self-reported)		
24-26 years	45	9		None	86	17
27 years or older	28	6		Eye-related (e.g., myopia, dry eye, etc.)	148	30
Gender				Musculoskeletal (e.g., cervical pain, back pain)	136	27
Male	210	42		Neurological (e.g., migraine, tension headache)	87	17
Female	288	58		Other	41	8
Year of studies				Primary device for smartphone use		
1st year MBBS	98	21		Smartphone	210	42
2nd year MBBS	220	44		Laptop/Computer	142	28
3rd year MBBS	127	25		Tablet	146	29
4th year MBBS	37	7		Daily internet connectivity		
Final year MBBS	16	3		Less than 2 hours/day	41	8
Residence				2-4 hours/day	156	31
Hostel/On-campus accommodation	209	42		5-8 hours/day	210	42
Living with family	289	58		More than 8 hours/day	91	18

Note. f = frequency, % = percentage; CVS = Computer Vision Syndrome; NDI = Neck Disability Index; HIT = Headache Impact Test; Values are presented as N (%), N = 498; No statistical comparisons were performed for demographic variables in this table

### Assessment of normality for key study variables

Table 2 presents the results of the normality tests for the study variables. Both the Shapiro-Wilk and the Kolmogorov-Smirnov tests were employed. The p-values of all variables (Smartphone Addiction Scale - Short Version (SAS-SV), Computer Vision Syndrome Questionnaire (CVS-Q), Neck Disability Index (NDI), and Headache Impact Test (HIT-6)) were not significant (p > 0.05), which implied that the distribution of the variables did not significantly differ between normality and non-normality. These results indicate that all the variables in the study are typically distributed, and thus, the subsequent statistical tests can be conducted using parametric tests.

**Table 2:** Tests of normality for study variables (N = 498)

Variables	Kolmogorov-smirnov statistic	df	p	Shapiro- wilk statistic	df	p
Smartphone addiction scale-short version (SAS-SV)	0.022	498	0.200	0.998	498	0.684
Computer vision syndrome questionnaire (CVS-Q)	0.027	498	0.200	0.996	498	0.412
Neck disability index (NDI)	0.024	498	0.200	0.997	498	0.531
Headache impact test (HIT-6)	0.021	498	0.200	0.999	498	0.812

Note. Kolmogorov-smirnov test used lilliefors significance correction; df = degree of freedom; A p-value > 0.05 was considered statistically significant, indicating normal distribution; N = 498.

### Correlations Among Smartphone Addiction, Computer Vision Syndrome, Neck Disability, and Headache Impact

Table 3 presents Pearson correlation coefficients between smartphone addiction, computer vision syndrome (CVS), neck disability (NDI), and headache impact (HIT-6) in medical students. Smartphone addiction positively correlated with CVS (r = 0.229, p < 0.001), NDI (r = 0.147, p = 0.001), and HIT-6 (r = 0.088, p = 0.049), meaning that the higher the smartphone addiction, the higher the ocular, cervical, and headache-related symptoms. The positive correlations were also observed between CVS and NDI (r = 0.191, p < 0.001) and HIT-6 (r = 0.180, p < 0.001), indicating a positive relationship between neck disability and headache impact, as well as increased ocular strain. NDI correlated with HIT-6 moderately (r = 0.169, p < 0.001), which means that neck disability is linked to the higher impact of headache. On the whole, these findings reveal some critical positive correlations between smartphone addiction, ocular strain, cervical disability, and the severity of headaches in this sample.

**Table 3:** Pearson correlations between smartphone addiction, computer vision syndrome, cervical disability, and headache impact among medical students (N = 498)

Variables	1	2	3	4
Smartphone addiction scale (SAS)	—	—	—	—
Computer vision syndrome (CVS)	r = 0.229, t(496) = 5.25, p < 0.001	—	—	—
Neck disability index (NDI)	r = 0.147, t(496) = 3.31, p = 0.001	r = 0.191, t(496) = 4.32, p < 0.001	—	—
Headache impact test (HIT-6)	r = 0.088, t(496) = 1.96, p = 0.049	r = 0.180, t(496) = 4.07, p < 0.001	r = 0.169, t(496) = 3.81, p < 0.001	—

Note. Values represent pearson correlation coefficients (r) between continuous variables; N=498; *=p<0.05, **=p < 0.01, ***=p<0.001 was considered statistically significant.

### Comparison of Study Variables by Gender

Table 4 illustrates the difference between male and female medical students in terms of smartphone addiction, computer vision syndrome (CVS), neck disability (NDI), and headache impact (HIT-6). The female scores were much higher than the male scores in all variables: Smartphone Addiction Scale (M = 33.20 vs. 30.50, t = -4.85, p = 0.001, d = 0.556), CVS (M = 6.50 vs. 5.80, t = -4.90, p = 0.001, d = 0.557), NDI (M = 5.05 vs. 4.70, t = -4.55, p < 0.001, d = 0.729), and HIT-6 (M = 63.8 vs. 60.5, t = -5.12, p < 0.001, d = 0.638) Such results were showing that female students were more affected by smartphone addiction, ocular strain, neck disability, and headache impact as compared to their male counterparts, with moderate to large effect sizes in all measures.

**Table 4:** Group statistics and comparison of smartphone addiction, computer vision syndrome, neck disability, and headache impact by gender (N = 498)

Variable	Male (N=210; 42%) M ± S.D	Female (N=288; 58%) M ± S.D	t	p	Cl 95%		Cohen's D
					LL	UL	
Smartphone addiction scale (SAS)	30.50 ± 4.50	33.20 ± 5.10	-4.85	<0.001**	-3.80	-1.60	0.556
Computer vision syndrome (CVS)	5.80 ± 1.20	6.50 ± 1.30	-4.90	<0.001**	-0.97	-0.43	0.557
Neck disability index (NDI)	4.70 ± 0.45	5.05 ± 0.50	-4.55	<0.001**	-0.50	-0.20	0.729
Headache impact test (HIT-6)	60.5 ± 5.0	63.8 ± 5.3	-5.12	<0.001**	-4.55	-2.05	0.638

Note. Values are presented as mean ± standard deviation; Independent samples t-tests were conducted to compare participants of both genders; Group sizes are shown as N (%); Reported statistics include p-values, t-values, 95% confidence intervals (CI), and effect sizes (Cohen's d); A p-value < 0.001 was considered statistically significant, N = 498.

### Age-related differences in smartphone addiction, ocular strain, neck disability, and headache impact

Table 5 indicates the prevalence and severity of smartphone addiction, computer vision syndrome (CVS), neck disability (NDI), and headache impact (HIT-6) among four age groups of medical students. The mean SAS-SV scores (M = 33.5, SD = 4.5) were highest in the younger age group (N = 350, 70%), who reported being more addicted to smartphones than the older age groups (F = 4.92, p = 0.002, η^2^ = 0.029). The highest level of CVS severity was observed in the 21-23-year-old group (M = 6.6, SD = 1.3). In contrast, the age group of participants (27 years and above) recorded the highest neck disability (M = 5.1, SD = 0.6). The difference in headache impact was also observed to vary significantly between age groups, with the 21-23 years reporting the highest HIT-6 scores (M = 64.2, SD = 5.0). The results were statistically significant (p < 0.01) with small to moderate effect sizes (0.029-0.111), indicating age-related differences in the use of digital devices, ocular strain, and severity of cervical pain and headache in medical students.

**Table 5:** Prevalence and severity of age, computer vision syndrome, cervical disability, and headache impact among medical students (N = 498)

Variable	18-21 years (N = 350; 70%) M ± SD	21-23 years (N = 75; 15%) M ± SD	24-26 years (N = 45; 9%) M ± SD	≥27 years (N = 28; 6%) M ± SD	F(3,494)	p	η^2^
Smartphone addiction scale (SAS-SV score)	33.5 ± 4.5	31.5 ± 4.0	30.8 ± 3.8	30.5 ± 4.2	4.92	0.002**	0.029
CVS severity (CVS-Q score)	5.8 ± 1.2	6.6 ± 1.3	6.0 ± 1.1	5.9 ± 1.0	14.17	<0.001**	0.079
NDI severity (NDI score)	4.7 ± 0.4	4.8 ± 0.5	5.0 ± 0.5	5.1 ± 0.6	20.62	<0.001**	0.111
Headache impact (HIT-6 score)	60.0 ± 4.8	64.2 ± 5.0	62.5 ± 4.6	61.2 ± 4.4	14.20	<0.001**	0.079

Note. Data are presented as mean ± standard deviation (M ± SD); Group sizes are shown as N (%); CVS = Computer vision syndrome; NDI = Neck disability index; HIT = Headache impact test; One-way ANOVA was conducted to examine the effect; All comparisons were significant at p < 0.01; η^2^ represents partial eta-squared effect size.

### Predictors of computer vision syndrome, neck disability, and headache impact: Multiple linear regression analysis

Table 6 reveals that a higher score on smartphone addiction (β = 0.255, p < 0.001) was the most predictive of computer vision syndrome, followed by neck disability (β = 0.150, p < 0.001) and the severity of headaches (β = 0.165, p < 0.001). The younger age (β = -0.072, p = 0.038), female gender (β = 0.085, p = 0.024), decreased physical activity (β = 0.060, p = 0.028), and the presence of medical conditions also showed significant predictive value for the more severe symptoms. On the whole, the primary contribution was made in smartphone addiction and associated health factors, with lesser but significant effects of age, gender, lifestyle, and comorbidities.

**Table 6:** Multiple regression analyses predicting computer vision syndrome, neck disability, and headache impact from smartphone addiction, age, gender, and other covariates (N = 498)

Predictor	B	SE	β	t	p	LL 95% CI	UL 95% CI
Constant (Computer vision syndrome questionnaire)	42.850	1.950	-	21.97	< 0.001**	38.999	46.701
Smartphone addiction (SAS)	0.210	0.032	0.255	6.56	< 0.001**	0.147	0.273
Cervical disability (NDI)	0.095	0.025	0.150	3.80	< 0.001**	0.046	0.144
Headache severity (HIT-6)	0.215	0.052	0.165	4.13	< 0.001**	0.113	0.317
Age (years)	-0.920	0.440	-0.072	-2.09	0.038*	-1.786	-0.054
Gender (Female = 1)	1.250	0.550	0.085	2.27	0.024*	0.164	2.336
Avg. physical activity (per week)	0.210	0.095	0.060	2.21	0.028*	0.023	0.397
Existing medical conditions	0.310	0.120	0.085	2.58	0.010*	0.074	0.546

Note. Multiple linear regression was conducted to identify predictors. Scores; Values include unstandardized coefficients (B), 95% confidence intervals (CI), standard error (SE), standardized beta coefficients (β), and p-values; *=p < 0.05, **=p < 0.01, ***=p < 0.001was considered statistically significant, N = 498.

### Regression analysis of smartphone addiction and other factors on CVS, NDI, and HIT-6

**Figure 1:**
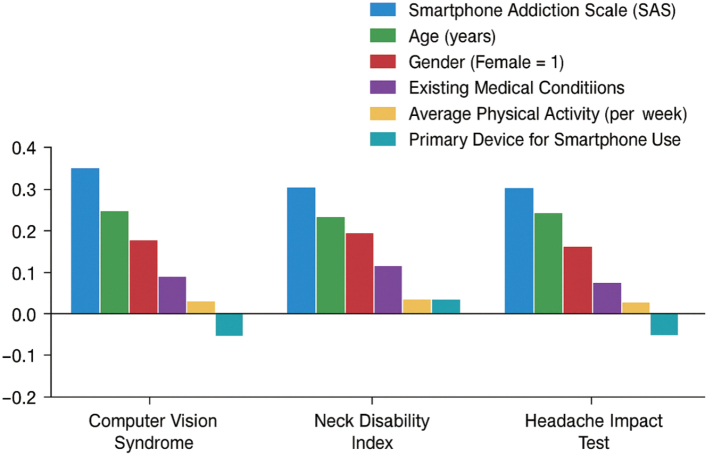
Standardized regression coefficients of smartphone addiction and demographic/behavioral factors predicting Computer Vision Syndrome, Neck Disability Index, and Headache Impact Test scores (N = 498)

Figure 1 indicates that smartphone addiction was the most significant predictor of computer vision syndrome, neck disability, and headache impact, followed by age and, finally, female gender. Alterations in medical conditions were also a risk factor, and physical activity showed a minor protective influence. There was little impact from using smartphones as the primary device.

## Discussion

This paper examined the relationship between problematic smartphone use (PSU) and physical health outcomes (i.e., oculomotor strain, cervical disability, and headache burden) among medical students. Our findings indicated that increased severity of smartphone addiction was significantly related to computer vision syndrome. A comparable result was also found in a previous study, where prolonged smartphone use was associated with refractive errors and dry eye disease, indicating the impact of excessive screen exposure on visual health issues^18)^. In our study, there was a weak but significant correlation between smartphone addiction and neck disability. A study also reported similar results, noting a moderate positive correlation that further indicates the association between long-term smartphone use and musculoskeletal issues^19)^. In our research, smartphone addiction was significantly and weakly associated with headache impact. This is evidenced by a survey, in which the addiction group expressed more prominent symptoms and more severe effects on everyday life, indicating that excessive smartphone use could exacerbate the burden of headache^20)^.

Our research revealed a strong correlation between computer vision syndrome and neck disability. Similar findings were observed in a comparative study, in which participants with persistent neck pain demonstrated higher scores in CVS, indicating the two-way relationship between visual strain and cervical issues^21)^. Students experiencing more severe headaches in our study had a higher CVS symptom. This finding is consistent with prior results, which indicate that headache burden is promoted by ocular discomfort and dry eye associated with prolonged screen use^22)^. In our research, a strong relationship was found between the impact of neck disability and headache. This is supported by the literature, which suggests that cervical musculoskeletal impairments may involve trigeminal-cervical mechanisms in increasing headache severity. Consequently, neck dysfunction may be a contributing factor to the headache burden^23)^.

The results of our study revealed that female students scored much higher on smartphone addiction than their male counterparts, in line with previous studies. The use of multimedia and social networking had a greater influence on female undergraduates^24)^. In our study, females displayed more intense Computer Vision Syndrome symptoms as compared to males, suggesting that they are more vulnerable to eye strain and other associated visual discomforts. This finding aligns with previous studies that suggest female gender is a risk factor for CVS^25)^. In our research, we discovered that female students scored significantly higher on neck disability scores than the men, which was as expected, given the findings of a cohort that showed women were more likely to receive disability pensions due to musculoskeletal disorders. This confirms the existence of significant gender disparities in neck disability^26)^. We have discovered that female students had greater headache impact than male students, similar to migraine studies in which women have more prolonged and more severe headaches and more symptoms. These results support the existence of gender disparities in the burden of headache^27)^.

In our study, the younger students reported more smartphone addiction than the older students, which aligns with previous research that indicated older age at the initial use of smartphones was linked with increased scores on addiction. It implies that younger age categories may be more susceptible to smartphone addiction, but recent usage patterns also contribute to this trend^28)^. In our study, the severity of CVS was found to be higher in students between 21-23 years of age, which coincides with a study that found a positive correlation between the longer the duration of computer use, the more severe the CVS. These results indicate that the more people are exposed to digital screens during early adulthood, the greater the severity of CVS^25)^. Our results suggest that older adults experience greater neck disability, with older students reporting higher scores in the NDI, which is in line with age-adjusted baseline NDI scores. This reinforces the idea that neck disability increases with age even in younger adults, which underscores the value of preventive intervention at an early age^29)^. We found that the impact of headaches was highest among students between the ages of 21 and 23 years, as research has indicated that there are age-related differences in the intensity of headaches and their symptoms. This suggests that the early adulthood phase might be a critical phase for increasing the headache burden, especially in women^27)^.

Based on the correlation results, the regression analysis supports the view that high smartphone addiction is an independent predictor of the intensity of Computer Vision Syndrome. Smartphone overuse harms visual health^18)^. Similarly, the regression analysis reveals that neck disability and headache severity are significant predictors of CVS, with the importance of cervical strain and ocular symptoms associated with prolonged screen exposure^21, 22)^. In addition, younger students and females were more predisposed to report higher CVS severity, indicating that age and sex are significant variables that affect the prevalence and outcome of CVS^25)^. In line with recent literature, lifestyle factors, such as physical activity, can influence the severity of CVS. Our results suggest that engaging in physical activity at least once a week (or more frequently) is loosely associated with increased CVS symptoms, possibly due to concurrent screen exposure and ergonomic factors^30)^. In line with our results, other researchers have reported that students with underlying medical or visual impairments, including those who use framed lenses, have higher CVS severity^31)^. It implies that comorbid health conditions can exacerbate the predisposition to visual strain and other cardiovascular symptoms, which require specific preventive interventions among these populations.

### Limitations and future directions

Several limitations should be acknowledged. First, this study employs a cross-sectional design, which means it cannot establish any causal relationships between smartphone addiction and health status. Reverse causality cannot be excluded; pre-existing eye or musculoskeletal problems could also predispose students to experience more strain while using a smartphone. Second, the data were obtained from self-reported questionnaires, and the findings may be subject to recall bias or social desirability bias. Third, the convenience sampling from a few medical colleges in Lahore may limit the generalizability of the study findings to medical students in different regions or a younger cohort. Fourth, complicating features, such as the degree of screen brightness, the ergonomics of the postures adopted during study, or workload/symptomatology and psychological status, were not investigated in detail.

Longitudinal or experimental design studies will be necessary to elucidate the causal pathways and provide clarity on the temporal sequence between smartphone use and its impact on physical health. Objective measures, such as screen-time tracking applications, posture observation, or ophthalmological assessments, could be included in studies alongside self-reports. Recruitment from different institutions and non-medical students would enhance generalizability. Additionally, intervention studies (such as digital hygiene training, ergonomic awareness programs, or exercise interventions) should investigate methods to reduce smartphone strain.

## Conclusion

This research found that there were considerable relationships between problematic smartphone use and oculomotor strain, cervical disability, and headache impact among medical students. These risks were further increased by female gender, young age, low physical activity, and pre-existing medical conditions. These results underscore the importance of raising awareness and implementing preventive measures, including promoting healthy digital lifestyles, teaching ergonomics, and engaging in regular physical activity. Targeting the prevention of problematic smartphone use is necessary not only to protect the physical well-being of students but also their academic performance and long-term health.

## Author contributions

NAS, JM, and SY conceived and designed the study. CI and DM provided critical input during the study design and supervised data collection. FJ, HH, AN, and SM contributed to data acquisition and data entry. GKC and TES performed statistical analyses and data interpretation. RA assisted in manuscript drafting and reference organization. NAS and JM critically revised the manuscript for important intellectual content. All authors read and approved the final version of the manuscript.

## Conflicts of interest statement

The authors declare that there are no conflicts of interest.
